# Hierarchy: enhancing performances beyond limits

**DOI:** 10.1093/nsr/nwaa249

**Published:** 2020-09-28

**Authors:** Clément Sanchez

**Affiliations:** Chimie de la Matière Condensée de Paris, UMR 7574, Collège de France, CNRS, UMPC Université Paris 06, Sorbonne University, PSL Research University, France; Chemistry of Ultra-Divided Matter at USIAS, University of Strasbourg Institute for Advanced Study, France

Living organisms, for their survival and reproduction, have evolved to produce complex hierarchical three-dimensional structures ranging from nanometers to meters with extraordinary and unusual sets of properties. Over the last 20 years, hierarchically porous materials that replicate complex living structures have attracted intense attention from both the academic and industrial worlds. A large variety of domains from biotechnology, biomedicine, catalysis, photocatalysis, optics, sensing, energy conversion and storage, and separation processes to cell therapy have a fervent interest in hierarchically porous materials [1–8]. We have witnessed a great evolution in the synthesis methodology and applications of hierarchically porous materials and a series of research works indeed break new ground in the field [4–14]. The most recent research from two research groups that are very active in the field brings new breakthroughs and merits particular highlighting.

**Figure 1.**
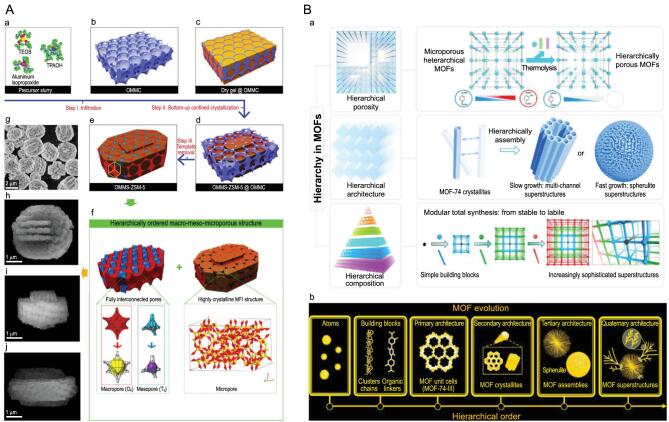
(A) Synthesis and structure of hierarchical ordered macro-meso-microporous zeolite single crystals. (a–e) Schematic illustration of the synthesis route. (f) Schematic illustration of the hierarchically ordered macro-meso-microporous structure in one zeolite single crystal. (g) SEM images of hierarchically ordered macro-meso-microporous zeolite single crystals. (h–j) SEM images of an individual crystal from three directions. Adapted with permission from refs [10] and [11]. Copyright 2020 Elsevier. (B) Hierarchically structured metal organic frameworks (MOFs) (a) from three different perspectives: hierarchical porosity, hierarchical architecture and hierarchical composition. (b) The hierarchy order in MOFs. Adapted with permission from ref. [12]. Copyright 2020 American Chemical Society.

The first to be mentioned is the work on micron-sized zeolite single crystals featuring hierarchical intracrystal interconnected ordered macro-meso-microporosity made by Su's group, the pioneers of the field, which is considered as one of the most important milestones of the field [3,10,11]. It is well known that as a size- and shape-selective catalyst, zeolites are widely used in petroleum and fine chemicals processing. However, their small micropores severely hinder molecular diffusion and are sensitive to coke formation. Hierarchically porous zeolite single crystals with fully interconnected, ordered and tunable multimodal porosity at macro-, meso- and micro-length scale, like in leaves, offer the ideal solution to minimize the diffusion limitations and the energy consumption of reactions in each of the zeolite crystals and to improve the zeolite catalyst efficiency and industrial catalytic and separation process performances. However, their synthesis remains highly challenging. Inspired by natural hierarchical structures, for the first time, they successfully synthesized a bio-inspired zeolite single crystal with a fully interconnected and highly ordered intracrystalline macro-meso-microporous hierarchy in all directions, as in leaves (Fig. 1A). Such unique hierarchically porous architecture within one single zeolite crystal with high thermal and hydrothermal stability and excellent mechanic strength can maximize the rate of intracrystalline diffusion of reactants and products, the accessibility to active sites and thus the catalytic efficiency of each of the zeolite single crystals. This leads to a significant reduction in coking and deactivation rate, outstanding catalytic activity and selectivity, and highly improved catalyst lifetime in methanol-to-olefins [10], bulky molecules cracking [10,11] and Friedel-Crafts alkylation of benzene with benzyl alcohol [11] reactions. Their *in situ* bottom-up confined crystallization strategy with regard to the hierarchically porous zeolite single crystal is highly universal and has been extended to the successful synthesis of other zeolitic materials, including ZSM-5, Beta, TS-1 and SAPO-34. It is envisioned that the utilization of this type of single-crystalline zeolite reactor in various reactions and procedures can lead to a revolution in industrial processes.

Furthermore, they have revealed the underlying physical principles of optimal nature systems by revisiting Murray's law and developing a generalized Murray's law (Equation (1)) via taking the mass variation and constant surface substance exchange into account during the mass transportation:

(1) \begin{equation*} \gamma _0^\alpha = \frac{1}{{1 - X}}\,\,\sum\limits_{i = 1}^N {\gamma _i^\alpha } , \end{equation*}

where the exponent α (2 or 3) is dependent on the type of transfer, and *X* is the ratio of mass variation during mass transfer in the parent pores [12].

This is the first time a physical structure of one complex porous material can be expressed by a quantitative mathematic expression. Following this generalized Murray's law, it is possible to establish the quantitative relationship between pore diameters of different length levels, and thus to guide the synthesis of bio-inspired hierarchically porous materials with maximized mass transport properties and minimized energy consumption.

Another milestone of the field in recent years is the research from Zhou's group on the hierarchy in metal-organic frameworks [13,14]. They found that in sequence-controlled hierarchical MOFs, the building blocks are placed in a specific order within a lattice, which strongly influences the molecular and macroscopic properties of framework materials (Fig. 1B) [13]. They developed a linker labilization strategy to create hierarchically porous structures with an integration of micropores, mesopores and windows that connect them throughout the crystal. The beneficial effect of such a structure on the adsorption properties and catalytic performances was demonstrated and promises a wide range of potential applications in guest adsorption/separations, heterogeneous catalysis, drug delivery and sensing [14].

Materials mimicking natural hierarchical structures at multiple length scales represent a promising approach to enhancing performance far beyond what can be achieved using composite structures, to add new functionalities and to adapt to special requirements. The generalized Murry's law can be the first impetus to the development of a general law of hierarchy for the design of materials with enhanced performances beyond limits.
